# Modular tipping points: How local network structure impacts critical transitions in networked spin systems

**DOI:** 10.1371/journal.pone.0292935

**Published:** 2023-11-14

**Authors:** Daniel Reisinger, Raven Adam, Fabian Tschofenig, Manfred Füllsack, Georg Jäger

**Affiliations:** Institute of Environmental Systems Sciences, University of Graz, Graz, Styria, Austria; The Hong Kong University of Science and Technology, CHINA

## Abstract

Critical transitions describe a phenomenon where a system abruptly shifts from one stable state to an alternative, often detrimental, stable state. Understanding and possibly preventing the occurrence of a critical transition is thus highly relevant to many ecological, sociological, and physical systems. In this context, it has been shown that the underlying network structure of a system heavily impacts the transition behavior of that system. In this paper, we study a crucial but often overlooked aspect in critical transitions: the modularity of the system’s underlying network topology. In particular, we investigate how the transition behavior of a networked system changes as we alter the local network structure of the system through controlled changes of the degree assortativity. We observe that systems with high modularity undergo cascading transitions, while systems with low modularity undergo more unified transitions. We also observe that networked systems that consist of nodes with varying degrees of connectivity tend to transition earlier in response to changes in a control parameter than one would anticipate based solely on the average degree of that network. However, in rare cases, such as when there is both low modularity and high degree disassortativity, the transition behavior aligns with what we would expected given the network’s average degree. Results are confirmed for a diverse set of degree distributions including stylized two-degree networks, uniform, Poisson, and power-law degree distributions. On the basis of these results, we argue that to understand critical transitions in networked systems, they must be understood in terms of individual system components and their roles within the network structure.

## Introduction

**Fig 1:**
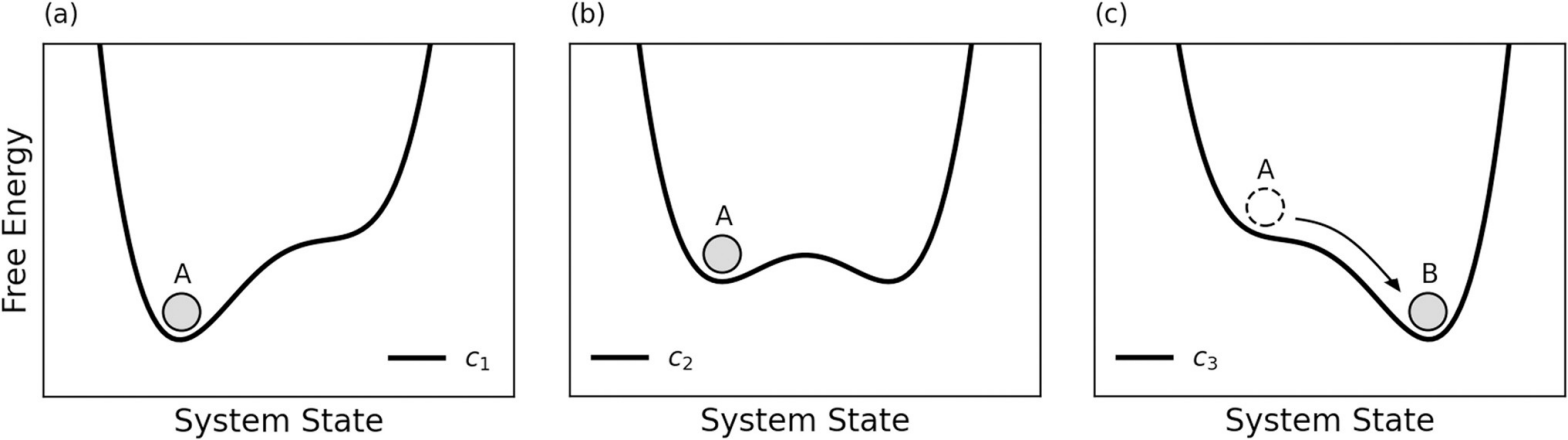
Shows a system’s critical transition from a stable state A to an alternative stable state B. The solid curved line represents the stability landscape of a system in which a ball, initially resting in state A, is driven towards state B as changes in the system’s control parameter *c* alter the stability landscape of the system.

Many systems are subject to abrupt and often irreversible regime shifts. One type of regime shift that holds significant importance in the study of complex systems is that of a critical transition. It describes a scenario in which a small change in the forces driving a system results in a large change in the state of that system [1]. To conceptualize a critical transition, it can be helpful to imagine the stability landscape of a system as a series of basins or potential wells in which a ball is rolling around [1] (see Fig 1). The ball represents the current state of the system and the depth of each well represents the degree of stability in the system. The resting position of the ball at the bottom of a well marks a stable state. Changes in the control parameter of a system result in changes in the stability landscape of that system. Qualitative changes in the stability landscape, that is, the emergence or disappearance of potential wells, can cause the ball to roll abruptly from its current position to an alternative potential well. This large positional change of the ball in response to the relatively small change in the control parameter of the system represents the system’s critical transition.

Critical transitions occur in a wide variety of real-life complex systems and prominent examples can be found in ecology [2–7], physics [8, 9], and social science [10–12], among others [13, 14]. To elaborate on some of these studies, an illustrative example from ecology is observed in shallow lakes, where the state of a lake abruptly shifts from a clear to a turbid state as the nutrient load of the lake is increased past a critical threshold [4]. In this case, the clear and the turbid states correspond to the ball resting in either one of the potential wells and the change of the lake’s nutrient load correspond to changes in the stability landscape of the system. Another example from the field of physics is the ferromagnetic transition where a ferrous material shifts from a magnetic to a non-magnetic state in response to changes in the material’s temperature or between a negative and a positive magnetic state in response to changes in an external magnetic field [8, 12, 15, 16]. A more recent example from sociology describes belief traps as a system of opinions, where the resilience of a belief system changes as the objective evidence for or against the belief is increased, creating a critical transition between two stable yet opposing opinions [10].

Critical transitions can vary significantly depending on the system being studied. In some cases, the entire system transitions as a unified whole. In other cases, the system transitions in cascades, where certain parts of the system transition prior to others. To understand when a system transitions as a unified whole and when in cascades, it is useful to take on a network perspective that focuses on the individual system components and the structural relationship between them. For example, the ferromagnetic system can be understood as a collection of individual spins connected to each other on a grid-like structure. The specific arrangement of these spins plays a crucial role in shaping the system’s transition behavior. Notably, modifying the structure such as through the introduction of impurities and defects in the lattice, leads to spatial variations in the coupling strength of the system [17]. As a result, this alteration distinctly affects the system’s resilience. Similarly, a social belief system can be conceptualized as individuals holding an opinion connected to each other in a social network structure. The structural properties of the social network including the placement and removal of influential individuals affect the resilience of communities, where isolated and tightly-knit groups may transition differently when faced with changes in external factors such as increasing evidence that contradicts current beliefs.

Often, researchers have to simplify a system by taking an aggregated system perspective, where local differences in the underlying network structure are averaged out or reduced to a mean-field representation. This simplification, however, can overlook crucial information about the true transition behavior of a networked system, where components may respond differently to external changes depending on their position within the network structure. For example, some system components may be embedded in highly resilient structures allowing them to transition quite late in response to changes in a control parameter, while others are embedded more weakly causing them to respond earlier to changes in a control parameter. In this context, it has been observed that changes in the network structure, such as modifying connections or altering node degrees, can significantly alter the onset of a system’s critical transition [15], and that peripheral components in the networked system appear to be more sensitive to changes in an external or control parameter [16]. In particular, previous results show that:

1. In a networked system, isolated components undergo a transition independently from each other [17], where components composed of low degree nodes transition earlier than components composed of high degree nodes [16].
2. A randomly networked system composed of multiple node degrees transitions as a unified whole and it transitions earlier in a response to changes in a control parameter than a comparable one-degree network of equal average degree, i.e. a random k-regular graph [15].

These results are intriguing as they demonstrate that the local arrangement of nodes can significantly impact a system’s transition behavior. They also give rise to additional questions, specifically:

1. How does the transition behavior of the networked system transform from an isolated component-wise transition to a unified transition, especially, one that responds earlier to what one would expect given the average degree of the network?
2. And under what, if any, structural requirements does a networked system composed of multiple node degrees, transition at the same control parameter value as a comparable one-degree network of equal average degree, i.e. a random k-regular graph?

To address these questions, this paper highlights a crucial but often overlooked aspect of critical transition behavior in networked systems: the modularity of the system’s underlying network structure. A system structure with low modularity indicates that connections between system components are relatively similar or uniform while a system structure with high modularity indicates differences and diversity among the components in terms of their connections and roles within the network. We argue that in highly modular systems, understanding the transition behavior requires considering the individual components and their roles within the network. Simply reducing the system to an aggregated state or mean-field is inadequate for accurately capturing the tipping behavior of a networked system. To support this claim, we conduct a comprehensive analysis of how controlled changes in the local network structure of a networked system affect its transition behavior. We place these findings within a spectrum from high to low modularity, providing a contextual framework for understanding the impact of local network structure on critical transition behavior.

## Methodology

In the following we describe how we simulate critical transitions on arbitrary networks, how we iteratively alter a system’s network structure through local rewiring, and how we measure the network’s modularity during this process.

### Simulating critical transitions with the Ising model

Critical transitions are simulated using a computational implementation of the Ising model [18]. Although the Ising model is typically linked to statistical physics and the investigation of ferromagnetic phase transitions, its simplistic working mechanism, which will be outlined in detail below, makes it applicable to a wide range of disciplines. For instance, in social science, the Ising model has been employed to study collective phenomena like opinion formation [19]. In ecology, it has been used to study spatial patterns in tree yield by considering root grafting and the coupling of trees [20]. Additionally, the Ising model has found utility in biology for examining brain functions, cancer behavior, and protein folding processes [21–24].

**Fig 2:**
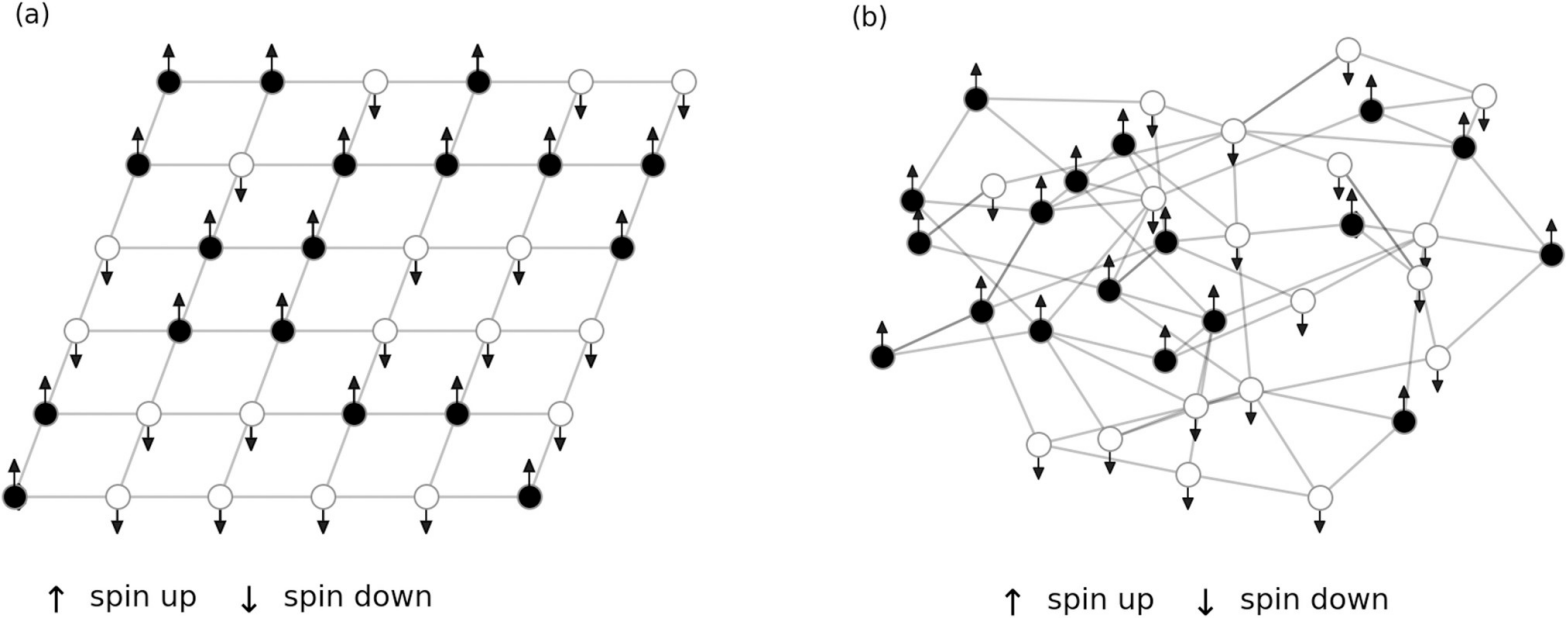
Ising model. (a) Standard 2D grid spinfield reproduced from [15]. (b) Spinfield on an arbitrary network. Every node in the network represents a spin that can be in two states, i.e. spin up or spin down. The spin states of a node’s immediate neighborhood determine the potential gain in energy for that node and thus its probability for flipping its spin.

The standard Ising model describes a field of spins connected in a grid-like structure (see Fig 2a). For our purposes, this constraint is loosened to include any given network structure (see Fig 2b).

**Fig 3:**
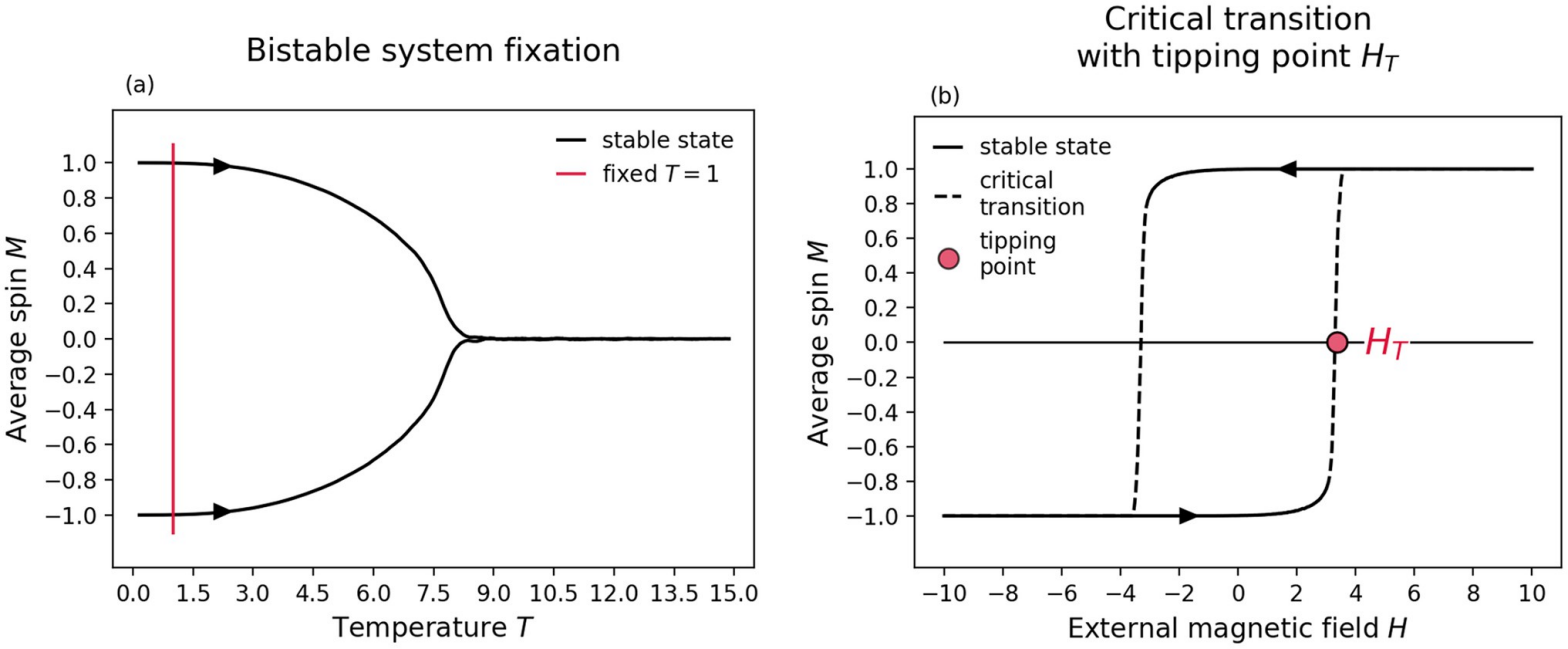
Critical transition in the Ising model run on an Erdős–Rényi random graph with *N* = 1000 and *q* = 0.008. (a) Shows how a networked system is locked in a bistable regime by fixing the temperature to *T* = 1. (b) Shows how the system critically transitions from *M* ≈ −1 to *M* ≈ 1 as the external magnetic field *H* is iteratively increased from *H* = −1 to *H* = 1. The tipping point *H*_*T*_ marks the value of the external magnetic field *H* for which half of the transition has occurred.

The nodes in the network represent spins which can be in one of two states. A node *i* with spin up is denoted as *s*_*i*_ = +1 and a node with spin down is denoted as *s*_*i*_ = −1. The edges in the network represent possible spin interactions. The specifics of the interaction between spins shall be referred to as the coupling mechanism of the Ising model, where every node in the network seeks a low energy state and flips its spin to minimize its potential gain in energy. This potential gain in energy is dependent on the node’s current spin and the spins of its neighboring nodes. It is given by

$$\begin{array}{c} E_{i}=2s_{i}\left(G_{i}+H\right) \end{array}$$ (1)

where *s*_*i*_ is the spin of a single node, *G*_*i*_ is the sum over all spin states in the direct neighborhood of node *i*, and *H* is an external magnetic field. A node flips its spin

$$\begin{array}{c} \text{if}\;E_{i}\leq 0\;\text{or}\;p<\text{exp}\left(-\frac{E_{i}}{T}\right) \end{array}$$ (2)

where *p* is random real number in the open interval [0, 1), and *T* is the internal temperature of the system. To simulate one iteration of the Ising model, all spins in the network are selected in random sequential order and updated according to the above equation. The critical transition, i.e. a first-order phase transition, is induced by iteratively varying the external magnetic field *H* of the system and fixing the temperature *T* so that the system is locked in a bistable state (see Fig 3). The system’s current state is expressed by its magnetization *M* which is calculated as the average spin over all nodes in the network.

$$\begin{array}{c} M=\frac{1}{N}\sum_{i}s_{i} \end{array}$$ (3)

where *N* is the number nodes in the networked system. In the ball and potential well scenario, the current state of the system or the average spin *M* of the spinfield correspond to the position of the ball and changes in the external magnetic field *H* correspond to changes in the stability landscape of the system. As the external magnetic field is increased or decreased over a certain threshold, the system critically transitions from one stable state to an alternative stable state (see Fig 3). Special emphasize shall be put on the tipping point *H*_*T*_ of the networked system, which marks the value of the external magnetic field *H* for which half of the transition to the alternative stable state has occurred.

### Altering the local network structure

The local network structure of a system is altered such that the degree assortativity coefficient *r* of the network changes. Is is calculated using [25, 26].

$$\begin{array}{c} r=\frac{\sum_{xy}xy\left(e_{xy}-a_{x}b_{y}\right)}{\sigma_{a}\sigma_{b}} \end{array}$$ (4)

where *e*_*xy*_ is the fraction of edges that connect nodes of degrees *x* and *y*, *a*_*x*_ and *b*_*x*_ are the fraction of edges that start and end at vertices with degrees *x* and *y*, and *σ*_*a*_ and *σ*_*b*_ are the standard deviations of *a*_*x*_ and *b*_*y*_ [25].

The degree assortativity coefficient *r* describes the degree correlation in the network and is defined on the closed interval *r* ∈ [−1, 1]. A positive degree assortativity coefficient means that high degree nodes tend to be connected to other high degree nodes, and low degree nodes tend to be connected to other low degree nodes. This scenario is often observed in social networks such as actor collaboration networks where influential individuals are connected to other influential individuals, and solitary individuals are connected to other solitary individuals [25]. A negative degree assortativity coefficient means that high degree nodes tend to be connected to low degree nodes and vice versa. This scenario is observed in many biological and technological networks such as protein interaction networks, animal food webs, and power grid networks, among others [25].

To analyze how the extent of degree assortativity in a network affects the shape and onset of a system’s critical transition we iteratively rewire the network structure from degree assortative to degree disassortative. This is done following a degree sequence preserving rewiring procedure by [27]. Degree sequence preserving means that the degree sequence of the network is not altered. A node *i* with given degree stays that degree, i.e. *deg*(*i*) = const. Only its relative position in the network changes. The algorithmic implementation works as follows. Step 1: Select two random edges with four distinct nodes. Step 2: Sort the nodes from highest to lowest degree. Step 3: To increase the degree assortativity coefficient, create two new edges by linking the two highest degree nodes and the two lowest degree nodes together. To decrease the degree assortativity coefficient, create two new edges by linking the node with the highest degree to the node with the lowest degree and the two remaining nodes. Step 4: Accept new edges if they do not create multi-links, otherwise repeat the rewiring procedure by selecting two new random edges with four distinct nodes.

It is worth noting that a degree assortativity coefficient of 1 or -1 respectively, may not be achievable for most degree sequence due to an unbalanced amount of nodes with different degrees [28]. In extreme cases, for example, in power law degree distributions, the maximum achievable degree assortativity or disassortativity coefficient can be much lower than the theoretic interval boundaries of *r*.

### Measuring the structural diversity in the network

In addition to shifting the degree assortativity of the network, we keep track of the network’s modularity. The modularity *Q* is calculated on a community split obtained by Louvain community detection as well as Clauset-Newman-Moore community detection [26, 29]. It describes the extent to which a network can be grouped into clusters or communities and provides a way to identify cohesive subgroups or functional units within a network. A network with high modularity exhibits strong modular structure meaning that nodes in the network are organized into distinct and well-defined modules or communities. In contrast, low modularity refers to a network where the modular structure is weak or absent, meaning that nodes in the network are less likely to form distinct modules or communities. Instead, the connections between nodes are more evenly distributed.

We further keep track of the standard deviation over the edge-betweenness centralities of the network, denoted as EBC *σ*. This provides us with another measure of the network structure’s diversity. If edges perform similar roles to each other, the standard deviation over the edge-betweenness centralities will be low. Contrary, if edges perform different roles from each other, for example, with some edges forming important bridges between communities, the standard deviation over the edge-betweenness centralities will be high.

### Simulation setup

To analyze how changes in the local network structure impact the transition behavior of a networked system, we devise the following simulation setup that covers four degree distributions: (a) two-degree networks, (b) uniform degree distribution networks, (c) Poisson degree distribution networks, and (d) power-law degree distribution networks:

(a) Two-degree networks are constructed using the configuration model [30] applied to a degree sequence comprised of nodes with two distinct degrees. We investigate two-degree networks exhibiting a small degree discrepancy constructed from a degree sequence (3_1_, 3_2_, …, 3_*n*_, 6_1_, 6_2_, …, 6_*m*_) with *n* = 600 and *m* = 300, and two-degree networks exhibiting a larger degree discrepancy constructed from a degree sequence (4_1_, 4_2_, …, 4_*n*_, 12_1_, 12_2_, …, 12_*m*_) with *n* = 1200 and *m* = 400. The number of nodes *n* and *m* are chosen such as to ensure that a degree assortativity coefficient of -1 and 1 is achievable. The small degree discrepancy network has *N* = 900 nodes and an average degree 〈*k*〉 = 4. The large degree discrepancy network has *N* = 1600 nodes and an average degree of 〈*k*〉 = 6.
(b) Uniform degree distribution networks are constructed using the configuration model [30] applied to a degree sequence (2_1_, …, 2_*n*_, 3_1_, …, 3_*n*_, …, 10_1_, …, 10_*n*_) with *n* = 100 resulting in a network with *N* = 900 nodes and an average degree 〈*k*〉 = 6.
(c) Poisson degree distribution networks are constructed using the Erdős–Rényi model with *N* = 1000 nodes and a probability for edge creation of *q* = 0.008 resulting in an average degree 〈*k*〉 ≈ 8. The Erdős–Rényi model operates by considering a fixed number of nodes and adding edges between pairs of nodes independently with a given probability, creating a graph where each potential edge exists with a certain probability, resulting in a range of graph structures from sparse to dense.
(d) Power-law degree distribution networks are constructed using the Barabási–Albert model with *N* = 1000 nodes and the number of edges to attach from a new node to existing nodes *m* = 2, resulting in an average degree 〈*k*〉 ≈ 4. The Barabási-Albert graph generator constructs a graph by iteratively adding nodes with edges that preferentially connect to existing nodes with higher degrees, leading to a scale-free network characterized by a few highly connected nodes and many nodes with few connections.

The networks constructed as described above have a degree assortativity coefficient *r* approximately equal to 0. To change this coefficient, we perform a series of rewiring steps on the network. These steps involve modifying the connections in the network to either increase or decrease the degree assortativity following the previously outlined rewiring process by [27]. We perform a total of 10^6^ rewiring steps, aiming to achieve both assortative (connections between nodes with similar degrees) and disassortative (connections between nodes with different degrees) networks given the underlying degree sequence.

To understand the effect of local rewiring on the transition behavior of the networked system, we simulate critical transition curves. This involves running an Ising simulation on altogether 4000 rewired networks where networks are chosen from the range of networks between the highest and lowest degree assortativity achieved during the rewiring process.

With regards to details for the Ising mechanism, we construct a transition curve, i.e. an average magnetization timeseries, by initializing the networked system with all spins pointing down, i.e. *s*_*i*_ = −1. By fixing the temperature *T* = 1 such that the system is locked in a bistable state, and then increasing the external magnetic field *H* iteratively from -1 to 1 until a transition is induced, we obtain the critical transition curve (as in Fig 3). Note that in each iteration, we go over all spins in random and sequential order and flip their spin according to the rules outlined previously. The tipping point *H*_*T*_, marks the control parameter value for which half of the transition has occurred. In our case, starting from an average magnetization *M* = −1, we define the tipping point *H*_*T*_ as the control parameter value *H* for which the average magnetization *M* of the system initially crosses *M* > 0 (as shown in Fig 3).

## Results

In the following we present our results for simple two-degree networks, i.e. networks constructed from degree sequences containing only two distinct degrees, and networks constructed from more diverse degree distributions, covering uniform, Poisson, and power-law degree distributions.

### Two-degree networks

**Fig 4:**
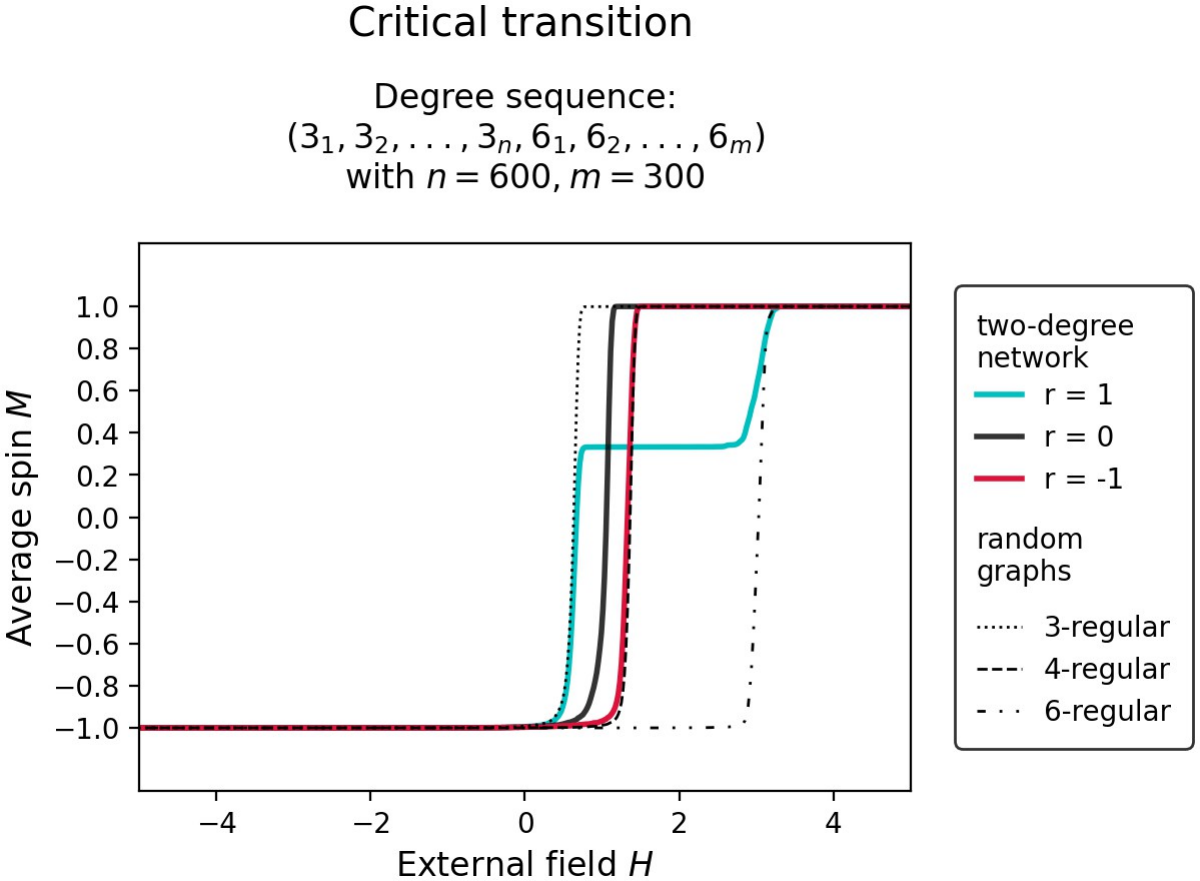
Critical transition from stable state *M* = −1 to *M* = 1 simulated in a two-degree network constructed from a degree sequence containing degrees 3 and 6. The red, black, and cyan solid lines corresponds to the transitions curves of the two-degree network with degree assortativity *r* ≈ −1, *r* ≈ 0, and *r* ≈ 1. The dotted and dashed lines are intended as reference lines and mark transition curves of random k-regular graphs or random one-degree networks, i.e. randomly configured networks constructed from a degree sequence containing only nodes of equal degree.

Fig 4 shows how the shape of a networked system’s critical transitions is influenced by the degree assortativity in the underlying network. It shows critical transition curves in a two-degree network with degree assortativity *r* ≈ −1, *r* ≈ 0, and *r* ≈ 1. We observe that a randomly configured two-degree network (Fig 4, *r* ≈ 0, black solid line) tips on average earlier than a randomly configured one-degree network of equal average degree, i.e. random k-regular network (black dashed line), confirming the results presented in a previous study which showed that randomly placed low-degree nodes disproportionally destabilize a networked system [15]. We also observe that a highly assortative two-degree network (Fig 4, *r* ≈ 1, cyan solid line), transitions in cascades, confirming the results presented in [16, 17]. This observation can be explained by considering that in a network with high degree assortativity, nodes with the same degree can be considered as almost independent networks where nodes of the same degree form coherent groups that are mostly disconnected from each other. Since groups with lower degrees transition earlier than those with higher degrees, the system transitions in cascades. Interestingly, a highly degree disassortative two-degree network (Fig 4, *r* ≈ −1, red solid line) tips at roughly the same control parameter value as the one-degree network of equal average degree, i.e. random k-regular graph(black dashed line). This suggests that the findings from [15] can be put further into context by considering local network structure to determine the limits to which tipping can occur earlier.

**Fig 5:**
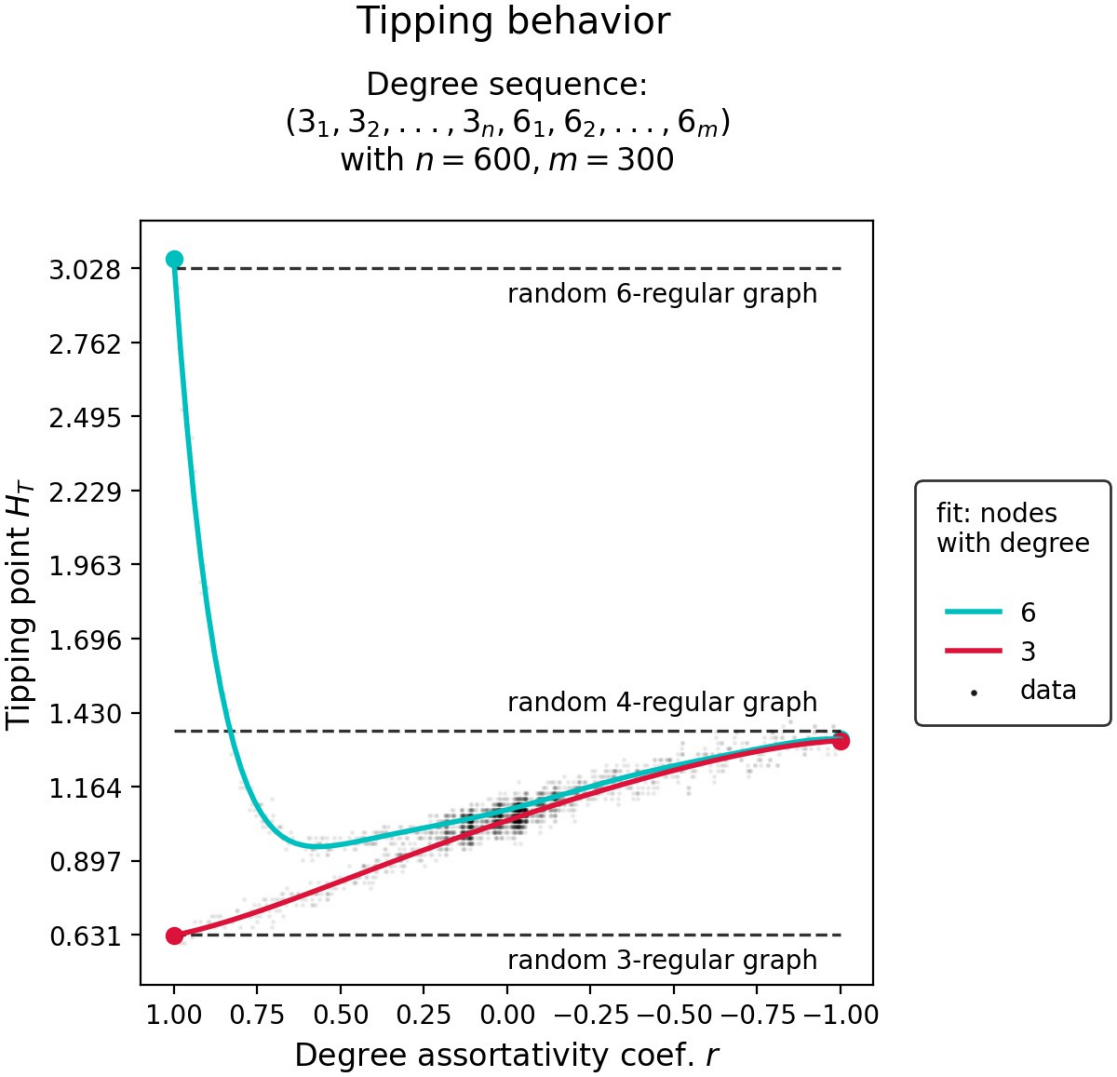
Tipping trajectories in a two-degree network. Shows change in tipping points *H*_*T*_, of nodes grouped by their degree, in response to changes in the degree assortativity coefficient *r* of the underlying network constructed from a degree sequence containing degrees 3 and 6.

Fig 5 shows the change in tipping points *H*_*T*_ of nodes grouped by their degree as the degree assortativity coefficient *r* of the network is varied between -1 and 1. With regards to our research questions where we ask how the transition behavior of a networked system transforms from a component-wise transition to a unified transitions, we find that components do not approach the unified tipping point symmetrically. As the degree assortativity of the networked system is decreased, the tipping point of the high node degree group shifts significantly more downwards than the tipping point of the low degree group shifts upwards. We further find that the speed of convergence toward unified tipping seems to be influenced by the discrepancy between individual degree groups. In particular, we observe fast convergence for smaller degree discrepancy networks such as the two-degree network constructed from nodes with degrees 3 and 6 (as in Fig 5) and slower convergence for larger degree discrepancy networks such as the two-degree network constructed from nodes with degrees 4 and 12 (not shown).

**Fig 6:**
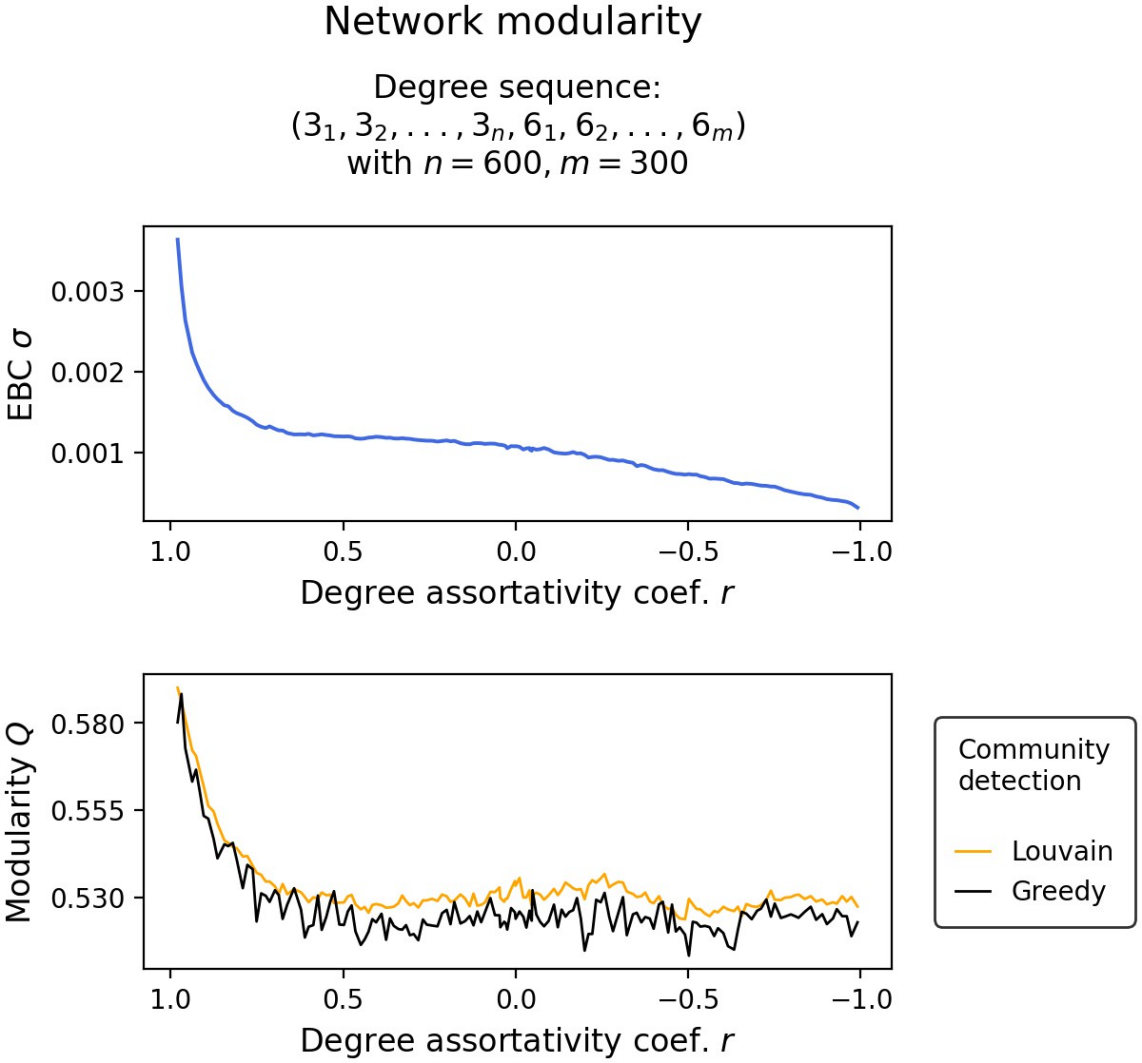
Shows changes in the standard deviation of edge-betweenness centralities EBC *σ* and modularity *Q* in response to changes in the degree assortativity coefficient *r* in a network constructed from a degree sequence containing degrees 3 and 6. The modularity *Q* is calculated on a community split obtained by Louvain community detection and Clauset-Newman-Moore greedy community detection.

To understand why the tipping points of a two-degree network and a one-degree network of equal average degree come together as the degree assortativity coefficient is decreased, it is imperative to consider the network’s structural diversity (see Fig 6. To reiterate, structural diversity describes the degree of similarity or uniformity in the connectivity patterns within a network. Here, it is expressed through the standard deviation of edge-betweenness centralities EBC *σ* and the modularity *Q* of the network. We see that, as the degree assortativity coefficient *r* is decreased, EBC *σ* and network modularity *Q* quickly drop, indicating that the network structure has becomes less diverse. In our two-degree networks, the highest level of structural homogeneity is achieved when the network exhibits complete degree disassortativity. This is particularly evident in the drop of the standard deviations of the edge-between centralities, EBC *σ*.

Intuitively, it makes a lot of sense that the level of structural diversity plays an important role here. If a small portion of the network contains significant information about the entire network, a global metric like the average degree would be sufficient to inform about the tipping point of the entire network. In contrast, if different parts of the network hold diverse and locally distinct structures, such that one part does not provide much insight into the rest of the network, then approximating the tipping point solely based on the average degree would not be informative. In such cases, the local differences in the network structure outweigh the global average, making it a less reliable indicator of the tipping point. Note that this represents an exceptional scenario, and as we will explore further, it is unattainable within alternative degree distributions.

### Other degree distributions

To provide a comprehensive view, we contextualize our previous results by presenting outcomes for alternative degree distributions. Visual representations depict the results for the uniform degree distribution, while for the Poisson and power-law degree distributions, which exhibit similar qualitative patterns, we provide a verbal discussion.

**Fig 7:**
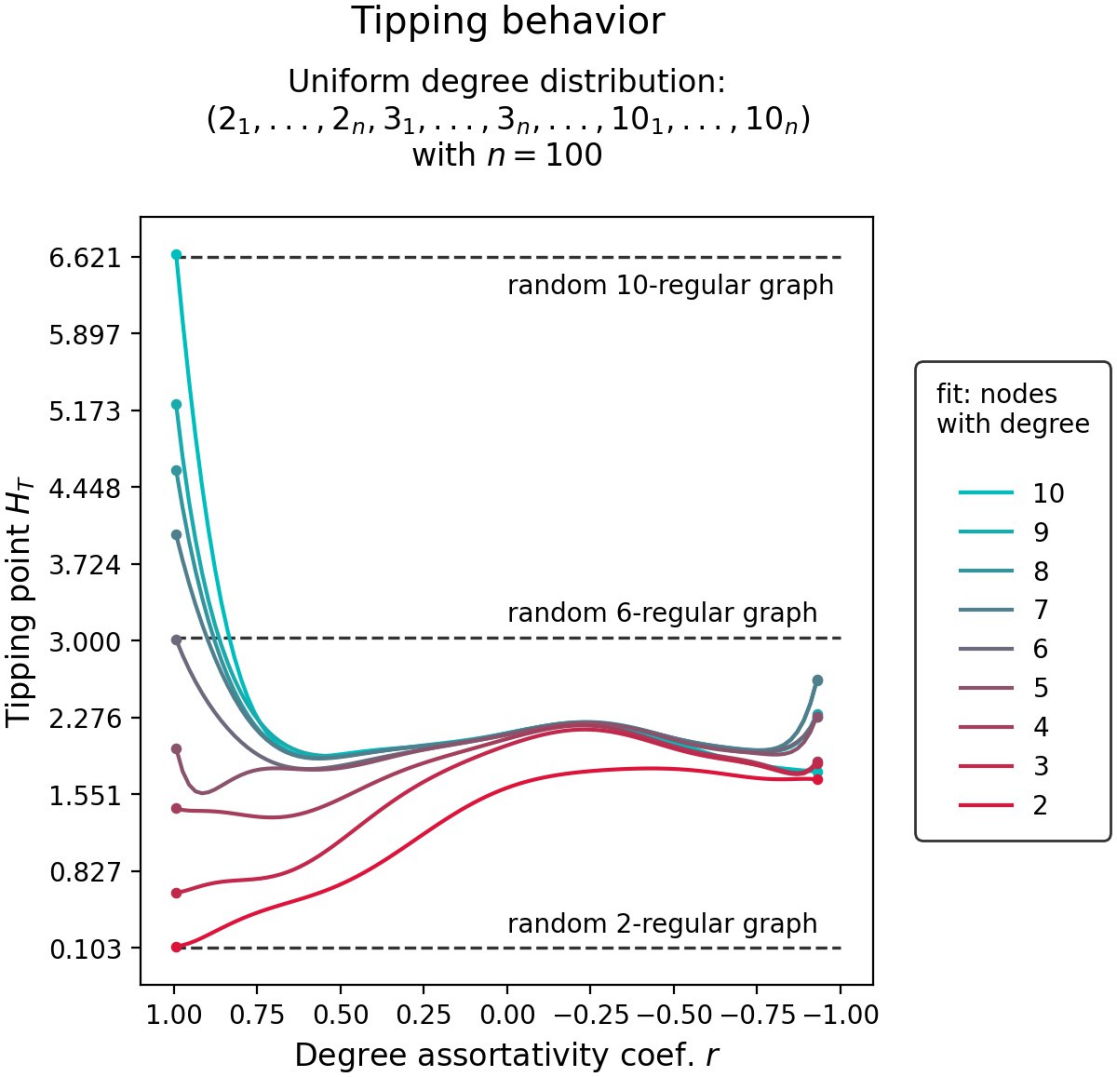
Tipping trajectory and structural homogeneity in a uniform degree distribution network. Left shows change in tipping points *H*_*T*_, of nodes grouped by their degree (see degree histogram) in response to changes in the degree assortativity coefficient *r*. Right shows change in structural homogeneity (standard deviation of edge-betweenness centralities EBC *σ* and modularity *Q*) in response to changes in the degree assortativity coefficient *r*.

**Fig 8:**
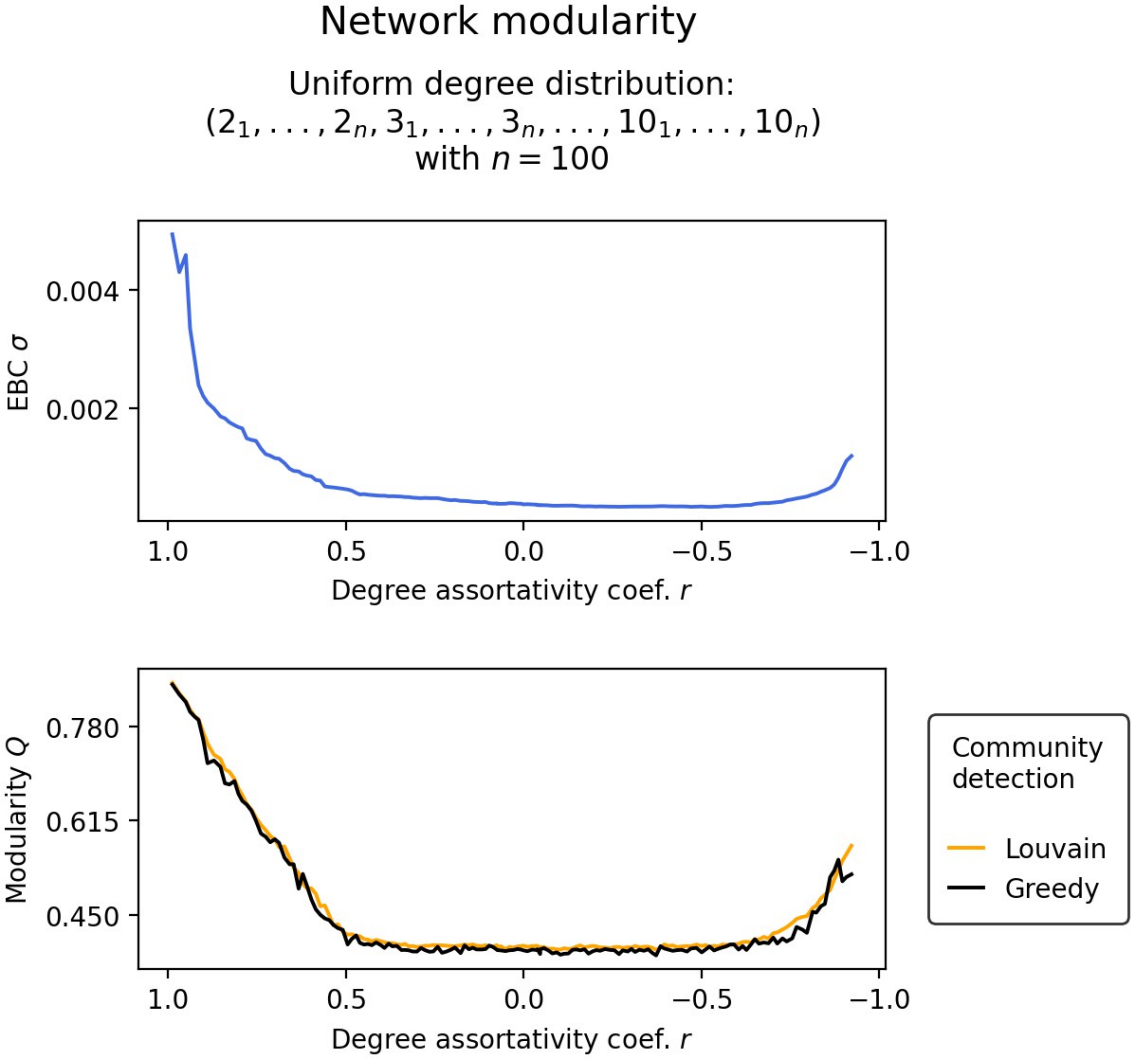
Shows changes in the standard deviation of edge-betweenness centralities EBC *σ* and modularity *Q* in response to changes in the degree assortativity coefficient *r* in a uniform degree distribution network. The modularity *Q* is calculated on a community split obtained by Louvain community detection and Clauset-Newman-Moore greedy community detection.

Fig 7 shows the change in tipping points *H*_*T*_ of nodes grouped by their degree as the degree assortativity coefficient *r* of a uniform degree distribution network is varied between -1 and 1. As the degree assortativity in the network is decreased to a random configuration, we observe that the network transforms from component-wise tipping to a more unified tipping, where the tipping point of high node degree groups shift significantly more downwards than the tipping point of low degree groups shift upwards. However, as the network is further driven into degree disassortativity, we observe, again, a tendency towards component-wise tipping. The reason for this lies in the diversity of the degree distribution. As the network becomes more disassortative, more extreme node pairs are formed (connecting nodes with the lowest degree to nodes with the highest degree, second lowest to second highest, and so on). As is shown in Fig 8, this leads to the network becoming more modular, which in turn causes the tipping trajectories to split apart.

Results are confirmed for both the Poisson and the power-law degree distribution networks. Note that, in the case of a power-law degree distribution network, the attainable range of degree assortativity after 10^6^ rewiring steps is considerably narrower. This arises due to nodes with extremely high degrees that cannot be effectively isolated from the rest of the network during the rewiring process.

## Discussion

This paper focused on understanding how changes in the local network structure affect the transition behavior of a networked system. Specifically, we simulated critical transitions in networked systems and investigated how controlled changes in the degree assortativity of the network influence the system’s tipping point. Below we briefly summarize our results:

1. Networked systems that exhibit low modularity transition as a unified whole, while networked systems exhibiting high modularity transition in cascades, confirming the results presented in [16, 17]. Cascading transitions can be attributed to the fact, that in modular network structures, groups of nodes form separate communities that transition independently of the rest of the network [17].
2. In two-degree networks, i.e. networks constructed from nodes with two distinct degrees, we find the following: Randomly configured two-degree networks (*r* ≈ 0) transition earlier than comparable one-degree networks of equal average degree. Degree assortatively configured two-degree networks (*r* ≈ 1) transition in cascades or in isolated node-groups of equal degree. Most notably, however, is that highly disassortatively configured two-degree networks (*r* ≈ −1) transition as a unified whole and close to the same control parameter value as a comparable one-degree network of equal average degree. This can be explained by the fact that in our two-degree networks, every low degree node can be linked exclusively to high degree nodes and vice versa. This creates an exceptionally low modular network structure where the average degree is able to capture most of the network’s information. Thus, tipping in degree disassortative two-degree networks that are have exceptionally low modularity can be well approximated by one-degree networks with the same average degree, i.e. random k-regular graphs.
3. In uniform, Poisson, and power-law degree distribution networks, we find the following: Again, randomly wired network configurations transition earlier than comparable one-degree networks of equal average degree, and degree assortatively wired networks transition in cascades or in isolated node-groups of equal degree. Interestingly, degree disassortatively wired networks also transition in cascades. This can be explained by the fact that in more complicated degree distributions, exceptionally low modularity is not achievable through high degree disassortativity. Nodes with the highest degree form an almost isolated group with nodes with the lowest degree, and nodes with the second highest degree form a group with nodes with the second lowest degree, and so on. The resulting network becomes increasingly modular and, thus, tips in cascades.
4. Finally, in all networked systems investigated, we find that isolated components do not approach the unified tipping point symmetrically. As the degree assortativity of the networked system is decreased, the tipping point of high node degree groups shift significantly more downwards than the tipping point of low degree groups shift upwards.

These results raise an interesting point with regards to the reducibility of a networked system. When a network has low modularity, it means that the nodes and connections are quite similar throughout the system. In case of exceptionally low modularity, which is, depending on the degree distribution, very difficult or even impossible to achieve, a simplified representation of the network, for example using a mean-field approximation, can be used to describe the entire system. The simplification works because in exceptionally low modular networks each part of the network is self-similar. Therefore, global network metrics like the average degree can be very informative about the whole system.

However, this approach has its limitations, especially when dealing with real-world network systems, which are often far from homogeneous. Many biological, technological, and sociological networks, for example, exhibit highly modular structures, with distinct communities or groups of nodes that may have different characteristics and connections. In such networks, relying solely on global network metrics or mean-field approximations may lead to inaccuracies in our understanding of the system’s transition behavior.

Another aspect in this regard is that networks with low modularity have similar resilience throughout the entire structure. This comes, however with certain cost. As has been shown in this study as well as in previous research by [15], well-mixed low degree nodes can disproportionally reduce the overall resilience of the system. On the other hand, in highly modular networks with distinct and tightly-knit communities, these communities can undergo transitions at different control parameter values. This means that some communities might transition sooner, while others might transition later. This creates, perhaps, a more flexible system that, as a whole, can exist longer in-between two stable states.

This highlights the importance of considering the individual responses of each component in the network structure rather than relying on the aggregated state of the system. To design and understand robust and resilient systems, further investigations into network structure of tipping systems are required. Understanding how different network structures and individual nodes influence the system’s behavior can provide valuable insights for creating more adaptive and stable networks. This is crucial for various fields, from social networks, to biological food webs and technological infrastructure, as it can help us build systems that can withstand and recover from disruptions more effectively.
